# Supplementary material for: Bispecific antibodies with broad neutralization potency against SARS-CoV-2 variants of concern
Source: bioRxiv. 2024 May 6:2024.05.05.592584. Preprint. [Version 1] doi: 10.1101/2024.05.05.592584 (PMC11100608; doi:10.1101/2024.05.05.592584)
Supplement: 1 [file NIHPP2024.05.05.592584V1-supplement-1.pdf]

bioRxiv preprint doi: https://doi.org/10.1101/2024.05.05.592584; this version posted May 6, 2024. The copyright holder for this preprint (which was not certified by peer review) is the author/funder, who has granted bioRxiv a license to display the preprint in perpetuity. It is made available under aCC-BY 4.0 International license.

Supplemental Materials

Bispecific antibodies with broad neutralization potency against SARS-CoV-2 variants of concern

Adonis A. Rubio, Viren A. Baharani, Bernadeta Dadonaite, Megan Parada, Morgan E. Abernathy, Zijun Wang, Yu E. Lee, Michael R. Eso, Jennie Phung, Israel Ramos, Teresia Chen, Gina El Nesr, Jesse D. Bloom, Paul D. Bieniasz, Michel C. Nussenzweig, Christopher O. Barnes

Supplemental Figure 1

Supplemental Figure 2

Supplemental Figure 3

Supplemental Figure 4

Supplemental Figure 5

Supplemental Figure 6

Supplemental Figure 7

Supplemental Figure 8

Supplemental Table 1

Supplemental Table 2

Supplemental Table 3

Supplemental Table 4

bioRxiv preprint doi: https://doi.org/10.1101/2024.05.05.592584; this version posted May 6, 2024. The copyright holder for this preprint (which was not certified by peer review) is the author/funder, who has granted bioRxiv a license to display the preprint in perpetuity. It is made available under aCC-BY 4.0 International license.

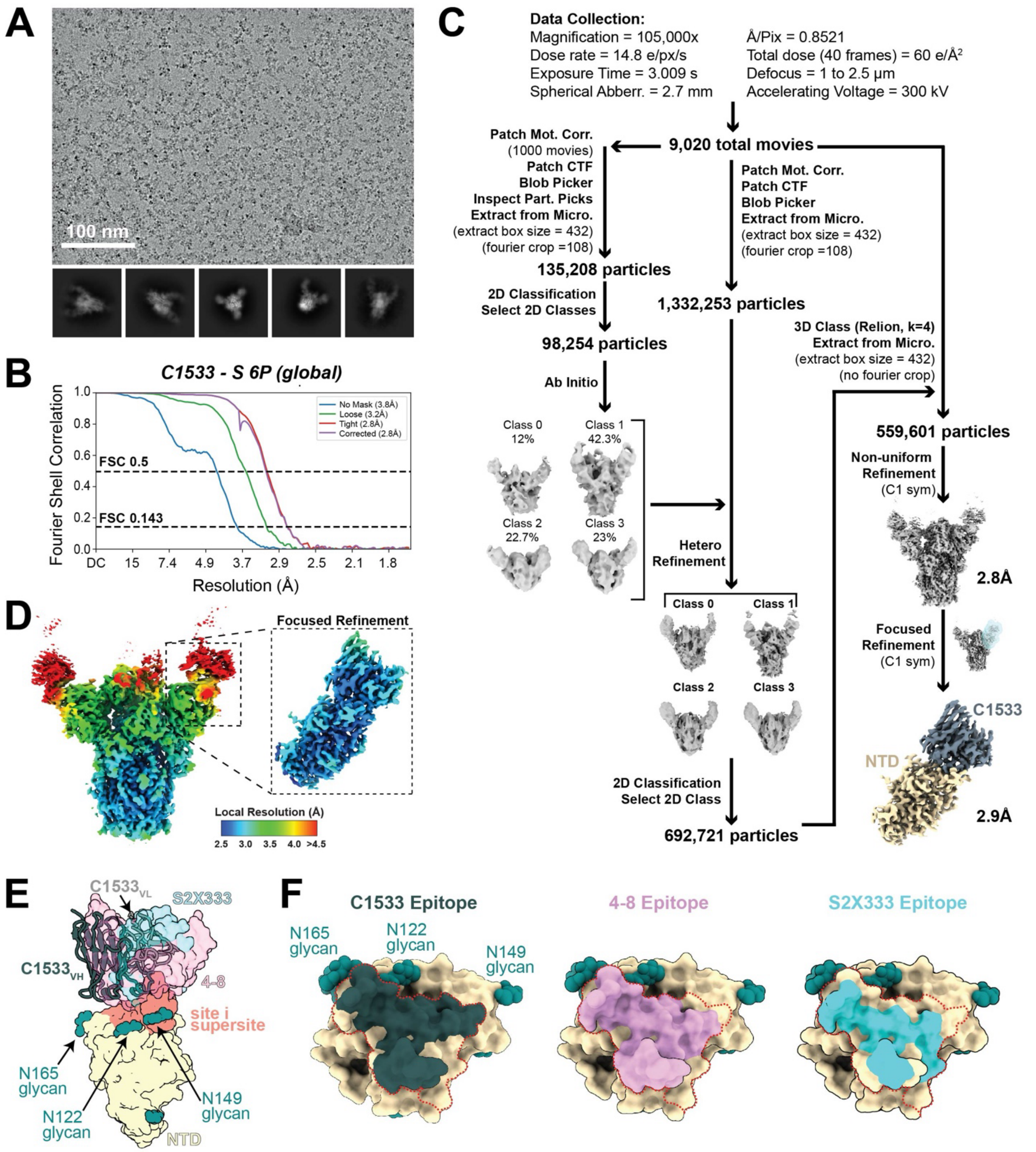

Supplemental Figure 1. C1533 Fab in complex with SARS-CoV-2 S 6P. Related to Figure 1. (A) Representative micrograph and 2D class averages selected from the total dataset of C1533-S 6P. **(B)** Gold-standard FSC plots for the C1533-S 6P global refinement. **(C)** Data collection and processing workflow. Final focused-refined map for C1533 variable domains (slate gray) bound

bioRxiv preprint doi: https://doi.org/10.1101/2024.05.05.592584; this version posted May 6, 2024. The copyright holder for this preprint (which was not certified by peer review) is the author/funder, who has granted bioRxiv a license to display the preprint in perpetuity. It is made available under aCC-BY 4.0 International license.

to the NTD subunit (wheat) to 2.9Å resolution is shown. **(D)** Local resolution estimations calculated in cryoSPARC for the C1533-S 6P global refinement and C1533$_{VhVl}$-NTD focused refinement (inset). **(E)** Superimposition of 4-8 (PDB: 8DLR, pink), S2X333 (PDB: 7LXW, cyan), and C1533 V_H (dark slate gray) and V_L (light gray) domains onto the SARS-CoV-2 Gamma P.1 NTD (PDB: 8DLR, wheat) after alignment on NTD residues 14-19, 67-69, 77-85, 100-122, 126-163, and 236-251 for a composite figure. **(F)** Surface rendering of SARS-CoV-2 Gamma P.1 NTD (PDB: 8DLR, wheat) with epitope footprints for C1533, 4-8, and S2X333 depicted, relative to the antigenic supersite (outlined in red).

bioRxiv preprint doi: https://doi.org/10.1101/2024.05.05.592584; this version posted May 6, 2024. The copyright holder for this preprint (which was not certified by peer review) is the author/funder, who has granted bioRxiv a license to display the preprint in perpetuity. It is made available under aCC-BY 4.0 International license.

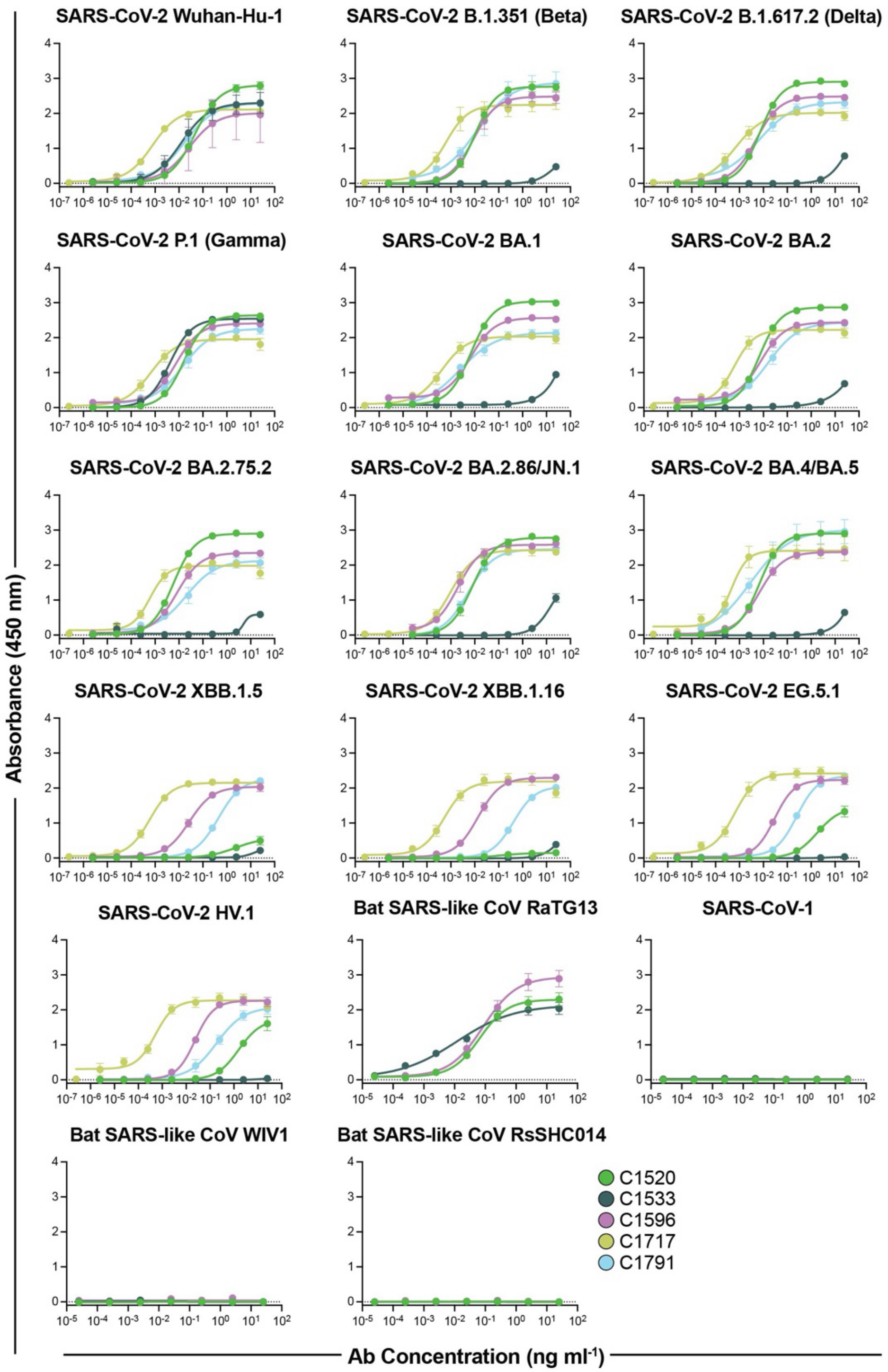

Supplemental Figure 2. ELISA curves for NTD-specific IgG antibodies binding to SARS-CoV-2 VOC and SARS-like NTD proteins. **Related to Figure 1.** ELISAs to compare C1520 (green), C1533 (dark slate gray), C1596 (magenta), C1717 (lime), and C1791 (cyan) binding to

bioRxiv preprint doi: https://doi.org/10.1101/2024.05.05.592584; this version posted May 6, 2024. The copyright holder for this preprint (which was not certified by peer review) is the author/funder, who has granted bioRxiv a license to display the preprint in perpetuity. It is made available under aCC-BY 4.0 International license.

directly-coated monomeric NTD protein constructs. Values represent the mean and standard error of the mean of three biological replicates (n=3).

bioRxiv preprint doi: https://doi.org/10.1101/2024.05.05.592584; this version posted May 6, 2024. The copyright holder for this preprint (which was not certified by peer review) is the author/funder, who has granted bioRxiv a license to display the preprint in perpetuity. It is made available under aCC-BY 4.0 International license.

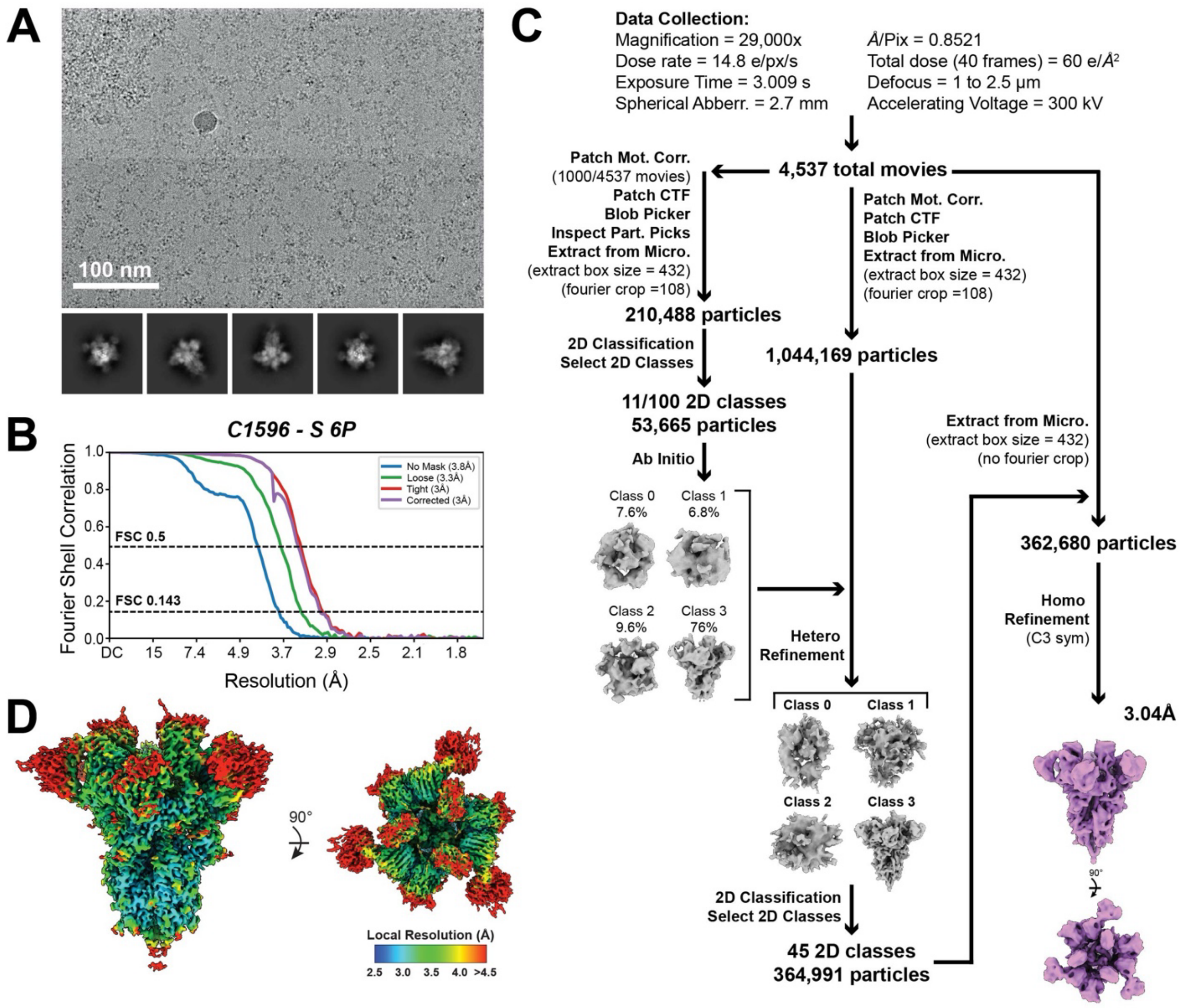

Supplemental Figure 3. Cryo-EM data collection and processing workflow for C1596-SARS-CoV-2 S 6P complex. Related to Figure 2. **(A)** Representative micrograph and 2D class averages selected from the total dataset for C1596-S 6P. **(B)** Gold-standard FSC plots for C1596-S 6P. (**C**) Processing workflow for C1596-S 6P dataset in CryoSPARC. (**D**) Local resolution estimations for C1596-S 6P.

bioRxiv preprint doi: https://doi.org/10.1101/2024.05.05.592584; this version posted May 6, 2024. The copyright holder for this preprint (which was not certified by peer review) is the author/funder, who has granted bioRxiv a license to display the preprint in perpetuity. It is made available under aCC-BY 4.0 International license.

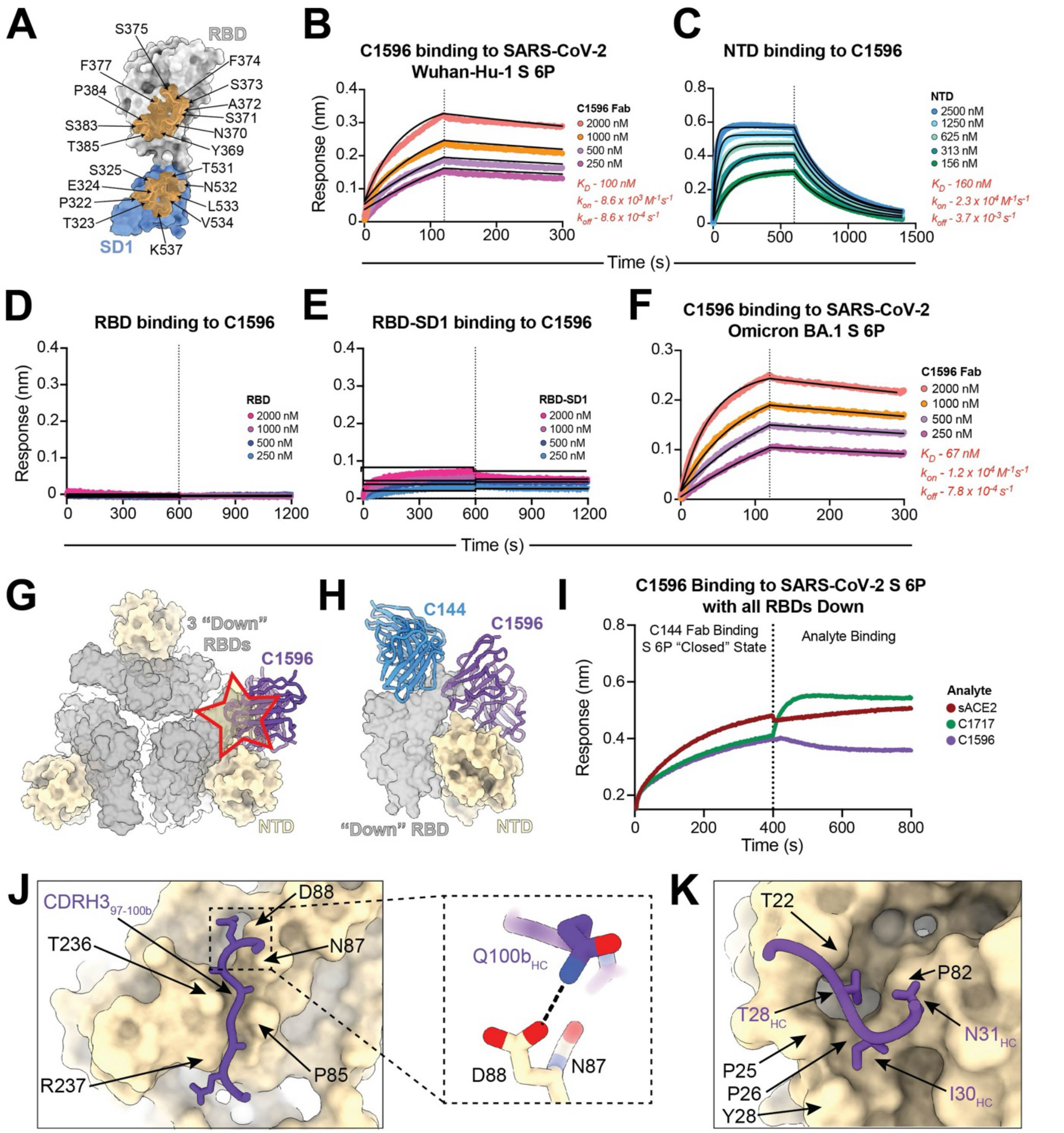

Supplemental Figure 4. Characterization of C1596 molecular interactions with spike S1 domains. Related to Figure 2. (A) C1596 epitope footprint (brown) outlined on the RBD (light gray) and SD1 (cornflower blue) domains, with interfacing amino acids denoted. **(B-F)** BLI binding kinetics data of C1596 Fab binding to immobilized SARS-CoV-2 Wuhan-Hu-1 S 6P trimer **(B)** or SARS-CoV-2 BA.1 S 6P trimer **(F)**, and monomeric NTD **(C)**, monomeric RBD **(D)**, or monomeric RBD-SD1 **(E)** binding to immobilized C1596 IgG. The concentrations of analyte are indicated on each panel. The vertical dotted lines indicate the transition between association

bioRxiv preprint doi: https://doi.org/10.1101/2024.05.05.592584; this version posted May 6, 2024. The copyright holder for this preprint (which was not certified by peer review) is the author/funder, who has granted bioRxiv a license to display the preprint in perpetuity. It is made available under aCC-BY 4.0 International license.

and dissociation phases. Fit curves are depicted as black solid lines. A K_D could not be determined in **(D)** or **(E)** due to the weak responses observed. **(G)** Modeling of C1596 V_H-V_L (purple) on the SARS-CoV-2 S 6P trimer (PDB: 7K90) in the "closed" state, with all three RBDs (light gray) in the down position. Steric clash of C1596 with an adjacent down RBD is depicted as a red and yellow star. **(H)** Modeling of C1596 V_H-V_L (purple) binding to the NTD (wheat) relative to C144 (blue) bound to an adjacent down RBD (PDB: 7K90), illustrating the lack of a clash between the two antibodies. **(I)** BLI experiment evaluating the ability of C1596 to bind S 6P when locked in the "closed" state. C144 Fab was bound to S 6P to conformationally lock the trimer in the "closed state", followed by a second association with C1596 Fab. Inclusion of ACE2 is depicted to confirm the "closed" state. Association with C1717 is depicted to illustrate the relative shift of an NTD-binding antibody that is not dependent on the RBD conformation. The vertical dotted line represents the transition between the two association steps. **(J)** Representation of C1596 CDRH3 residues contacting a groove in the NTD. Inset: Stabilizing contact with residues Q100b of the C1596 CDRH3 mediated by a potential hydrogen bond with the NTD. **(K)** Representation of C1596 heavy chain engaging a hydrophobic pocket on the NTD utilizing framework region 1 and CDRH1 residues.

bioRxiv preprint doi: https://doi.org/10.1101/2024.05.05.592584; this version posted May 6, 2024. The copyright holder for this preprint (which was not certified by peer review) is the author/funder, who has granted bioRxiv a license to display the preprint in perpetuity. It is made available under aCC-BY 4.0 International license.

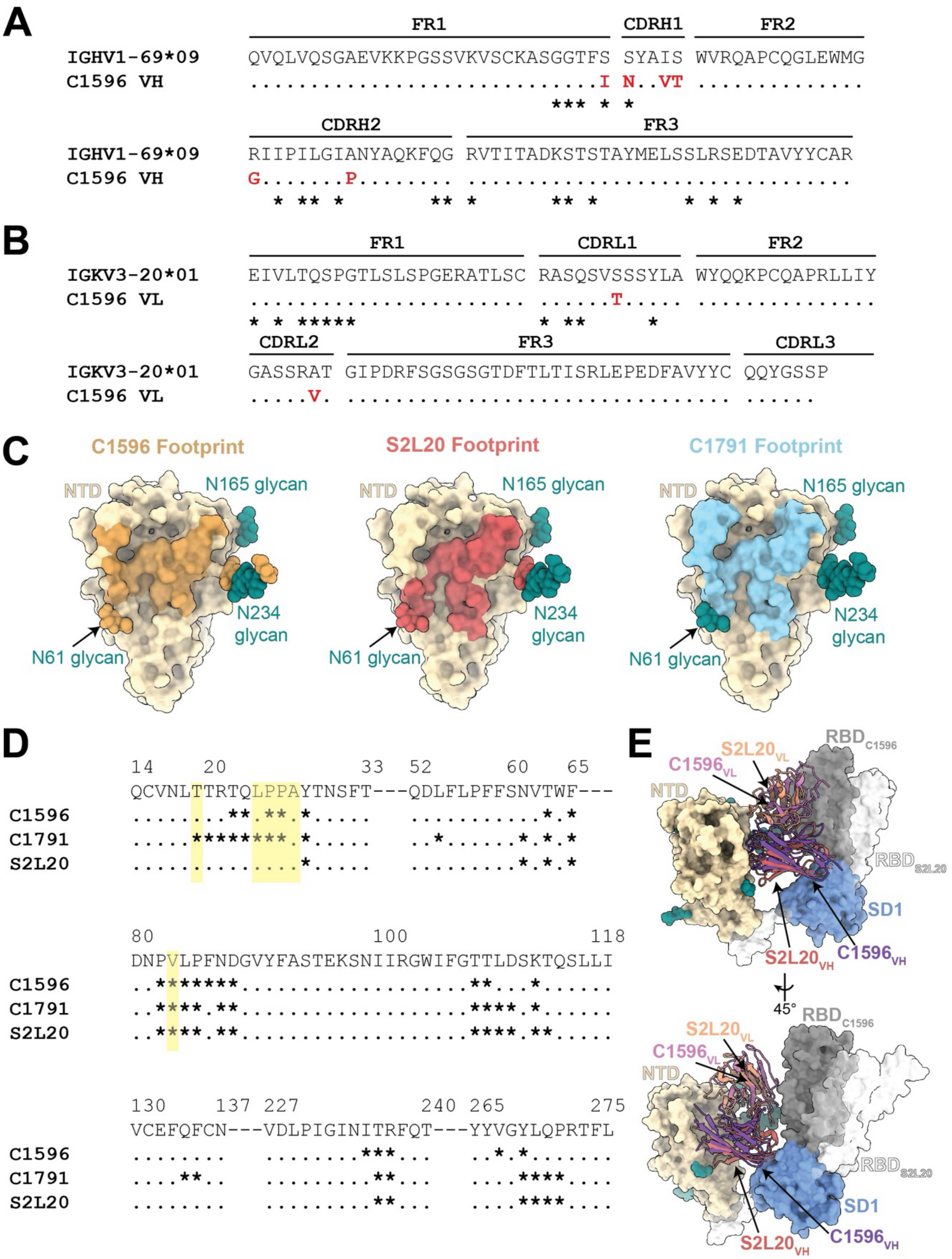

Supplemental Figure 5. C1596 binds the glycopeptidic NTD epitope shared by C1791 and S2L20. Related to Figure 2. (A-B) Amino acid sequence alignment of C1596 **(A)** V_H and **(B)** V_L to germline reference sequences determined by V-Quest/IMGT. Mutations from the germline

bioRxiv preprint doi: https://doi.org/10.1101/2024.05.05.592584; this version posted May 6, 2024. The copyright holder for this preprint (which was not certified by peer review) is the author/funder, who has granted bioRxiv a license to display the preprint in perpetuity. It is made available under aCC-BY 4.0 International license.

sequence are shown in red. Paratope residues of C1596 at the spike trimer interface are denoted with an asterisk. Framework regions, FR; and complementarity determining regions, CDRs, annotated using Kabat sequence numbering. **(C)** Surface rendering of SARS-CoV-2 Wuhan-Hu-1 NTD (wheat) with epitope footprints for C1596 (left, brown), S2L20 (middle, red), and C1791 (right, light blue) depicted. **(D)** Sequence representation of contacted NTD residues for C1596, C1791, and S2L20, depicted by asterisks. Sites where Omicron XBB.1.5 mutations overlap with NTD contacts are highlighted in yellow. **(E)** Overlay of V_H and V_L domains of C1596 (shades of purple) and S2L20 (PDB: 8GTQ; shades of red) after alignment on NTD residues 14-307, illustrating similar binding poses, but contrasting engagement with the RBD of the same protomer.

bioRxiv preprint doi: https://doi.org/10.1101/2024.05.05.592584; this version posted May 6, 2024. The copyright holder for this preprint (which was not certified by peer review) is the author/funder, who has granted bioRxiv a license to display the preprint in perpetuity. It is made available under aCC-BY 4.0 International license.

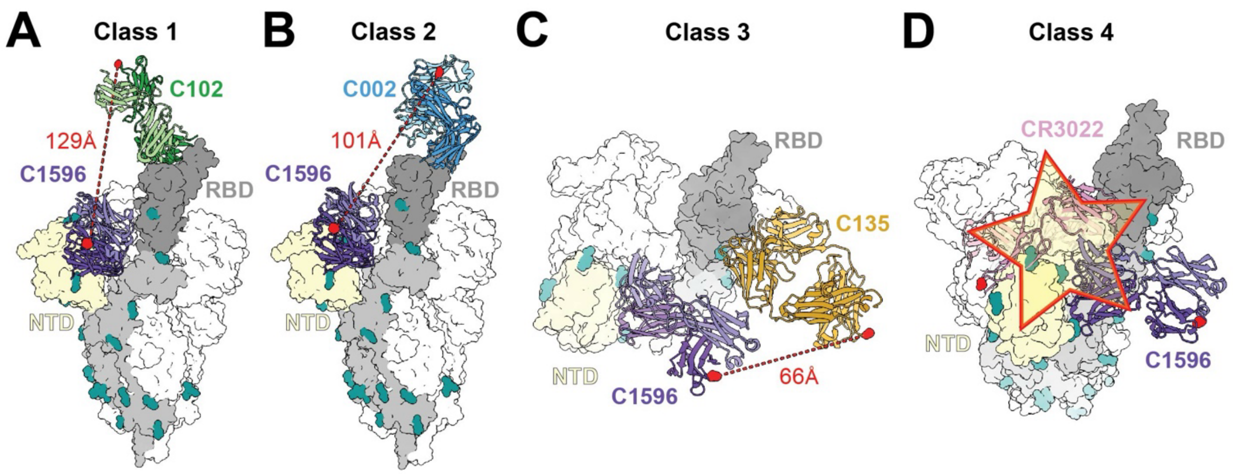

Supplemental Figure 6. Structural modeling of distances between C1596 and RBD antibodies for bispecific design. Related to Figures 2 and 3. (A-D) Measurement of Cα distance (red dotted line) between the C termini of C1596 CH_1 (purple) and the CH_1 domains of **(A)** C102 (class 1 RBD epitope), **(B)** C002 (class 2 RBD epitope), **(C)** C135 (class 3 RBD epitope), and **(D)** CR3022 (class 4 RBD epitope). **(A-D)** A representative full-length Fab domain model was created for C1596 by aligning the variable heavy chain of C105 (PDB: 6XCA) to the variable heavy chain of C1596, aligning on residues 1-120 at the Cα. NTD-C1596 Fab models were then aligned to the NTD of the SARS-CoV-2 S glycoprotein (PDB: 7T67), aligning on NTD residues 14-307 at the Cα. **(A)** The RBD-C102 Fab model (PDB: 7K8M) was aligned to the RBD of the SARS-CoV-2 S, aligning on RBD residues 331-529 at the Cα. **(B)** A representative full-length Fab domain model was created for C002 by aligning the variable heavy chain of the C002 Fab (PDB: 7K8O) to the variable heavy chain of the C002 VH-VL model (PDB: 7K8T), as described for the C1596 Fab modeling. The RBD-C002 Fab model was then aligned to the RBD of the SARS-CoV-2 S, as described in panel A. **(C)** A representative full-length Fab domain model was created for C135 by aligning the variable heavy chain of the C135 Fab (PDB: 7K8R) to the variable heavy chain of the C135 VH-VL model (PDB: 7K8Z), as described for C1596. The RBD-C135 Fab model was then aligned to the RBD of the SARS-CoV-2 S, as described in panel A. **(D)** The RBD-CR3022 Fab model (PDB: 6YLA) was aligned to the RBD of the SARS-CoV-2 S model, as described in panel A.

bioRxiv preprint doi: https://doi.org/10.1101/2024.05.05.592584; this version posted May 6, 2024. The copyright holder for this preprint (which was not certified by peer review) is the author/funder, who has granted bioRxiv a license to display the preprint in perpetuity. It is made available under aCC-BY 4.0 International license.

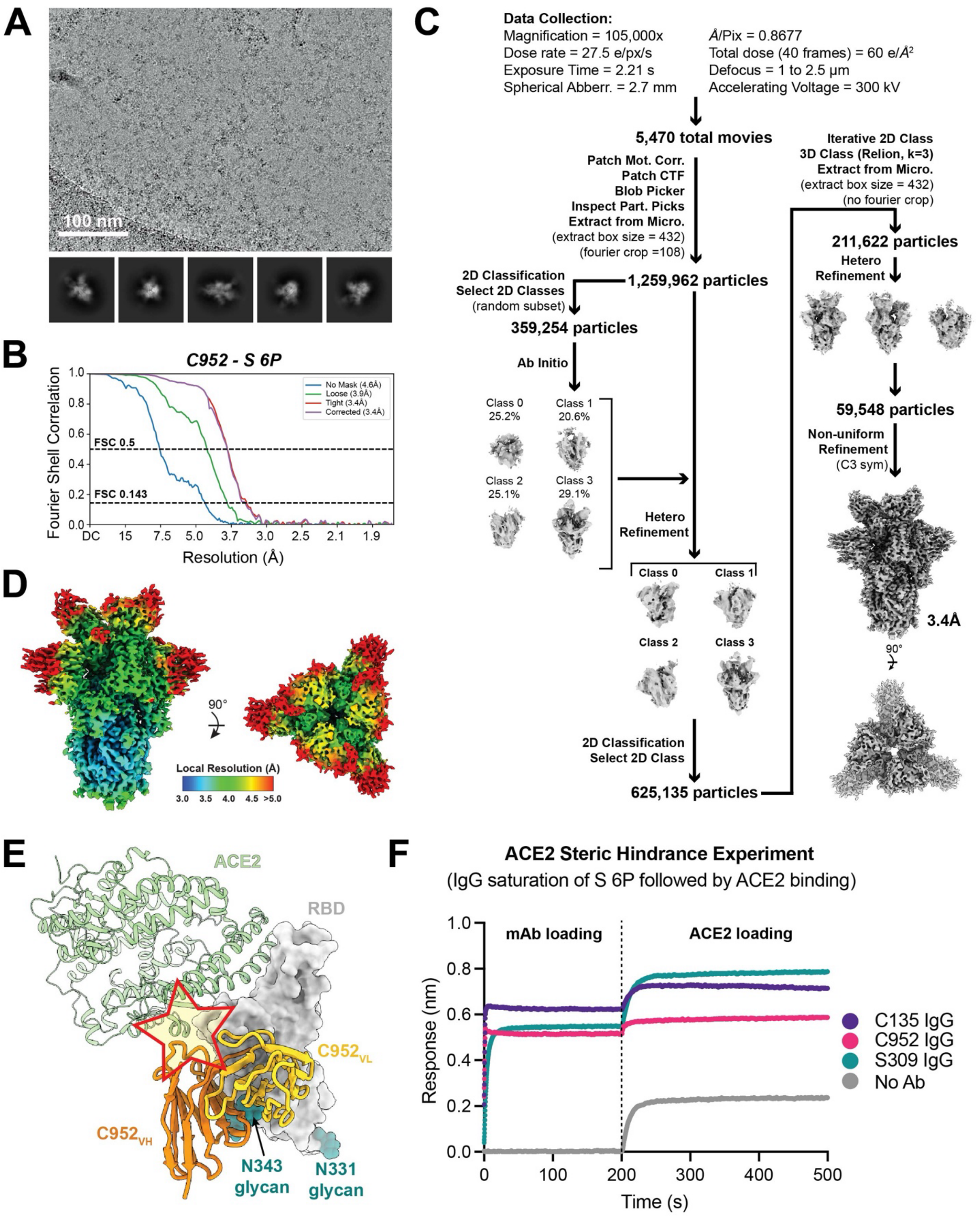

Supplemental Figure 7. Cryo-EM data collection and data processing workflow for C952 Fab in complex with SARS-CoV-2 S 6P. Related to Figure 3. (A) Representative micrograph

bioRxiv preprint doi: https://doi.org/10.1101/2024.05.05.592584; this version posted May 6, 2024. The copyright holder for this preprint (which was not certified by peer review) is the author/funder, who has granted bioRxiv a license to display the preprint in perpetuity. It is made available under aCC-BY 4.0 International license.

and 2D class averages selected from the total dataset of C952-S 6P. **(B)** Gold-standard FSC plots for the C952-S 6P global refinement. **(C)** Data collection and processing workflow. **(D)** Local resolution estimations calculated in cryoSPARC for the C952-S 6P global refinement. **(E)** Composite model of C952–RBD (shades of orange and gray, respectively) overlaid with soluble ACE2 (green; PDB 6M0J). Model was generated by aligning structures on 188 RBD Cα atoms. Potential clashes between ACE2 and C952 are highlighted by a yellow star. **(F)** mAb and ACE2 competition experiment by BLI. SARS-CoV-2 S 6P was immobilized on a streptavidin biosensor and saturated with either C135 (purple), C952 (pink), or S309 (green) mAb before dipping into a 1 μM solution of soluble ACE2 (as indicated by vertical dashed line). An ACE2 binding event (i.e., increase in y-axis response) indicates no competition for RBD binding between ACE2 and the corresponding mAb.

bioRxiv preprint doi: https://doi.org/10.1101/2024.05.05.592584; this version posted May 6, 2024. The copyright holder for this preprint (which was not certified by peer review) is the author/funder, who has granted bioRxiv a license to display the preprint in perpetuity. It is made available under aCC-BY 4.0 International license.

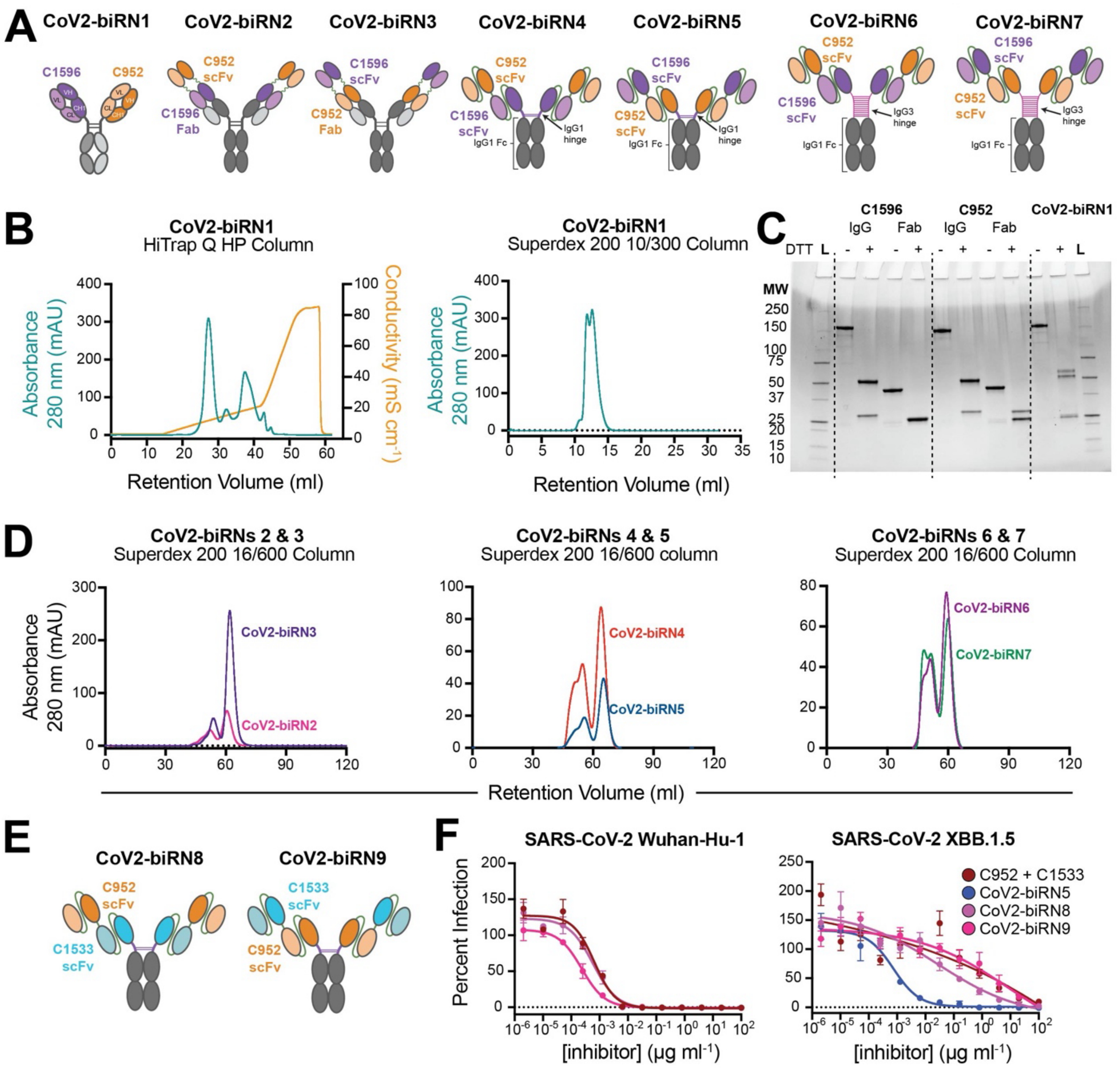

Supplemental Figure 8. CoV2-biRN construct design and protein purification workflow. Related to Figure 4. (A) Schematic representation of CoV2-biRNs 1-7. **(B)** Representative chromatograph of (left) anion exchange followed by (right) size exclusion chromatography of CoV2-biRN1. **(C)** Protein gel of CoV2-biRN1 after anion exchange and size exclusion with parental antibodies ran as a reference under non-reducing or reducing conditions (L; ladder). **(D)** Representative chromatographs of the size exclusion purification of CoV2-biRNs 2 to 7. **(E)** Schematic of C952-C1533 tandem scFv bsAb constructs (CoV2-biRN8 and CoV2-biRN9). **(F)** Representative neutralization curves for CoV2-biRN8 and CoV2-biRN9 against (left) SARS-

bioRxiv preprint doi: https://doi.org/10.1101/2024.05.05.592584; this version posted May 6, 2024. The copyright holder for this preprint (which was not certified by peer review) is the author/funder, who has granted bioRxiv a license to display the preprint in perpetuity. It is made available under aCC-BY 4.0 International license.

CoV-2 Wuhan-Hu-1 and (right) SARS-CoV-2 XBB.1.5 pseudoviruses. Data points represent the mean with standard error of the mean.

	C1520			C1533			C1596		
	k_{on} ($M^{-1}s^{-1}$)	k_{off} (s^{-1})	K_D (M)	k_{on} ($M^{-1}s^{-1}$)	k_{off} (s^{-1})	K_D (M)	k_{on} ($M^{-1}s^{-1}$)	k_{off} (s^{-1})	K_D (M)
SARS-CoV-2 D614G S 6P	*N.T.*	*N.T.*	*N.T.*	*N.T.*	*N.T.*	*N.T.*	8.62E+03	8.58E-04	9.954E-08
SARS-CoV-2 BA.1 S 6P	*N.T.*	*N.T.*	*N.T.*	*N.T.*	*N.T.*	*N.T.*	1.17E+04	7.77E-04	6.66E-08
Wuhan-Hu-1 NTD	1.59E+04	3.93E-04	2.48E-08	3.20E+04	5.47E-04	1.71E-08	3.02E+04	3.79E-03	1.25E-07
B.1.351 NTD	2.05E+04	5.55E-04	2.71E-08	*N.B.*	*N.B.*	*N.B.*	2.45E+04	3.46E-03	1.41E-07
B.1.617.2 NTD	3.00E+04	1.41E-03	4.70E-08	*N.B.*	*N.B.*	*N.B.*	2.43E+04	3.00E-03	1.24E-07
P.1 NTD	1.31E+04	3.01E-03	2.30E-07	4.31E+04	1.07E-03	2.49E-08	2.35E+04	6.63E-03	2.82E-07
BA.1 NTD	3.55E+04	4.18E-03	1.18E-07	*N.B.*	*N.B.*	*N.B.*	4.47E+04	3.94E-03	8.82E-08
BA.2 NTD	3.60E+04	5.14E-04	1.43E-08	*N.B.*	*N.B.*	*N.B.*	4.03E+04	2.76E-02	6.85E-07
BA.2.75.2 NTD	3.07E+04	5.69E-03	1.85E-07	*N.B.*	*N.B.*	*N.B.*	3.63E+04	2.20E-02	6.07E-07
BA.2.86/JN.1 NTD	1.96E+04	1.10E-03	5.63E-08	*N.B.*	*N.B.*	*N.B.*	7.61E+04	5.55E-04	7.29E-09
BA.4/BA.5 NTD	4.63E+04	5.28E-04	1.14E-08	*N.B.*	*N.B.*	*N.B.*	7.27E+04	4.90E-02	6.74E-07
XBB.1.5 NTD	*N.B.*	*N.B.*	*N.B.*	*N.B.*	*N.B.*	*N.B.*	9.90E+04	5.24E-02	5.29E-07
XBB.1.16 NTD	*N.B.*	*N.B.*	*N.B.*	*N.B.*	*N.B.*	*N.B.*	8.92E+04	5.27E-02	5.91E-07
EG.5.1 NTD	*N.B.*	*N.B.*	*N.B.*	*N.B.*	*N.B.*	*N.B.*	1.08E+05	4.43E-02	4.10E-07
HV.1 NTD	*N.B.*	*N.B.*	*N.B.*	*N.B.*	*N.B.*	*N.B.*	7.07E+04	3.91E-02	5.53E-07

	C952		
	k_{on} ($M^{-1}s^{-1}$)	k_{off} (s^{-1})	K_D (M)
Wuhan-Hu-1 RBD	2.78E+05	1.76E-03	6.35E-09
BA.1 RBD	3.01E+05	1.70E-04	6.27E-09
BA.2.75.2 RBD	2.93E+05	3.43E-04	1.73E-08
BA.2.86 RBD	1.41E+02	1.13E-01	8.00E-04
BA.4/BA.5 RBD	4.35E+05	3.58E-03	8.22E-09
XBB.1.1 RBD	*N.B.*	*N.B.*	*N.B.*
XBB.1.5 RBD	4.63E+04	4.60E-03	9.93E-08
BQ.1.1 RBD	*N.B.*	*N.B.*	*N.B.*
EG.5.1 RBD	1.60E+03	2.89E-01	1.80E-04
HV.1 RBD	3.94E+01	3.70E-02	9.39E-04
JN.1 RBD	3.66E+01	8.44E-02	2.31E-03

Supplemental Table 1. Binding kinetic data for monoclonal antibodies against SARS-CoV-2 VOC recombinant proteins. **Related to Figures 1 and 3.** N.B., no binding; N.T., not tested.

bioRxiv preprint doi: https://doi.org/10.1101/2024.05.05.592584; this version posted May 6, 2024. The copyright holder for this preprint (which was not certified by peer review) is the author/funder, who has granted bioRxiv a license to display the preprint in perpetuity. It is made available under aCC-BY 4.0 International license.

Contribution of SARS-CoV-2 S domains and glycans to the C1596 epitope

		Ab Interaction Breakdown (Å^2)		
**Domains**	**Area (Å^2)**	**Heavy Chain**	**Light Chain**	**Percentage**
NTD	754	661	15	59
RBD	291	0	285	23
SD1	174	237	0	14
S1 Glycans	67	13	52	5
Total Area	1286			

Contribution of C1596 Framework and CDR residues to C1596 paratope

	Heavy Chain		Light Chain	
**Region**	**Area (Å^2)**	**%**	**Area (Å^2)**	**%**
Framework 1	151	17	161	46
CDR1	62.9	7	191	54
Framework 2	0	0	0	0
CDR2	217	24	0	0
Framework 3	187	21	0	0
CDR3	294	32	0	0
Framework 4	0	0	0	0
Total Area	912		352	

Supplemental Table 2. C1596 epitope and paratope buried surface area. Related to Figure 2.

bioRxiv preprint doi: https://doi.org/10.1101/2024.05.05.592584; this version posted May 6, 2024. The copyright holder for this preprint (which was not certified by peer review) is the author/funder, who has granted bioRxiv a license to display the preprint in perpetuity. It is made available under aCC-BY 4.0 International license.

	C1533 SARS-CoV-2 NTD (local)	C1596 SARS-CoV-2 S 6P (global)	C952 SARS-CoV-2 S 6P (global)
**PDB**	**9BJ2**	**9BJ3**	**9BJ4**
**EMD**	**44627**	**44628**	**44629**
**Data collection conditions**			
Microscope	Krios G2	Krios G2	Krios G2
Camera	K3	K3	K3
Magnification	105,000x	29,000x	105,000x
Voltage (kV)	300	300	300
Dose rate (e^-/pixel/s)	14.8	14.8	27.5
Electron dose (e^-/Å ^{2})	60	60	60
Defocus range (µm)	1 - 2.5	1 - 2.5	1 - 2.5
Pixel size (Å)	0.8521	0.8521	0.8677
Micrographs collected	9,020	4,537	5,470
Micrographs used	8,904	4,241	5,067
Total extracted particles	1,332,253	1,044,169	1,259,962
Final refined particles	559,601	362,680	59,548
Symmetry imposed	C1	C3	C3
Nominal Map Resolution (Å)			
FSC 0.143 (unmasked/mask	2.88/2.87	3.04/3.01	3.61/3.41
**Refinement and Validation**			
Initial model	7LXY, 7COE, 6PZY	7K4N, 7R8O, 5I1L	73BO, 7N3E
Model Resolution (Å)			
FSC 0.143	2.8	3.04	3.4
Number of atoms	3,920	30,621	28,697
Protein	3,879	29,400	28,616
Ligand	41	1221	81
MapCC (global/local)	0.8/0.84	0.84/0.87	0.75/0.77
Map sharpening B-factor	107	115	100
R.m.s. deviations			
Bond lengths (Å)	0.002	0.005	0.004
Bond angles (°)	0.509	1.035	0.585
MolProbity score	1.78	1.6	1.84
Clashscore (all atom)	5.62	3.97	6.62
Poor rotamers (%)	2.6	0.28	1.97
Ramachandran plot			
Favored (%)	97.13	93.79	96.25
Allowed (%)	2.87	6.21	3.58
Disallowed (%)	0	0	0.17

Supplemental Table 3. Cryo-EM data collection and data processing statistics. Related to Figures 2 and 3 and Supplemental Figure 1.

	C952	C1596	C952 + C1596	CoV2-biRN1	CoV2-biRN2	CoV2-biRN3	CoV2-biRN4	CoV2-biRN5	CoV2-biRN6	CoV2-biRN7
**IC_{50} (ng ml^{-1})**										
Wuhan-Hu-1 D614G	1.44 ± 0.3	479 ± 29.5	1.64 ± 0.75	10 ± 1.33	3.52 ± 0.36	12.4 ± 2.6	3.1 ± 1.27	5.95 ± 1.15	2.8 ± 0.81	2.64 ± 1.33
BA.1	0.21 ± 0.22	334 ± 115	0.26 ± 1.6	2.65 ± 0.31	0.58 ± 0.14	4.86 ± 4.29	0.24 ± 0.39	0.42 ± 0.88	0.21 ± 0.23	0.47 ± 0.16
BA.2	0.423 ± 0.13	7,910 ± 13,600	0.378 ± 0.1	5.48 ± 9.75	0.269 ± 0.529	4.33 ± 9.57	0.991 ± 0.398	0.511 ± 0.229	0.546 ± 0.1	1.01 ± 1.0
BA.2.86	1,650 ± 414	79.9 ± 27.1	36.4 ± 15.6	184 ± 44.1	17.7 ± 17.1	39.2 ± 56.9	7.58 ± 3.15	3.03 ± 1.56	5.28 ± 2.66	5.39 ± 1.94
BA.4/BA.5	0.42 ± 0.1	1,180 ± 9,060	1.57 ± 24.1	19.6 ± 4.05	0.99 ± 0.1	10.2 ± 3.87	1.48 ± 0.45	0.44 ± 0.27	1.53 ± 0.54	1.57 ± 0.77
XBB.1.5	4,610 ± 2,360	12,800 ± 2,280	3,590 ± 781	5,430 ± 924	364 ± 51.9	1,280 ± 940	232 ± 51.2	2.91 ± 1.84	131 ± 73.2	3.51 ± 0.57
XBB.1.16	3,800 ± 3,790	8,770 ± 5,490	1,170 ± 495	3,360 ± 1,630	763 ± 804	1,120 ± 490	199 ± 57.1	4.73 ± 0.599	127 ± 133	3.64 ± 0.75
EG.5.1	5,510 ± 1,910	4,450 ± 5,890	4,310 ± 3,980	4,490 ± 5,140	153 ± 107	479 ± 259	76.5 ± 29.5	4.6 ± 2.81	68.3 ± 8.82	1.15 ± 0.25
HV.1	5,970 ± 2,420	18,200 ± 8,740	1,320 ± 103	3,630 ± 735	212 ± 40	1,260 ± 206	261 ± 167	5.26 ± 1.74	53 ± 26.4	3.52 ± 0.55

	C952	C1596	C952 + C1596	CoV2-biRN1	CoV2-biRN2	CoV2-biRN3	CoV2-biRN4	CoV2-biRN5	CoV2-biRN6	CoV2-biRN7
**IC_{90} (ng ml^{-1})**										
Wuhan-Hu-1 D614G	12.6 ± 10.8	2,970 ± 1,950	15.6 ± 1.52	26.1 ± 3.31	9.75 ± 3.76	58.3 ± 21.8	13.7 ± 2.38	38.6 ± 6.93	11.4 ± 0.38	14.7 ± 4.74
BA.1	7.29 ± 2.64	3,890 ± 2,150	3.37 ± 1.68	25.9 ± 6.95	3.83 ± 3.59	52 ± 64.8	2.92 ± 3.87	11 ± 15.4	18.6 ± 10.6	8.14 ± 1.7
BA.2	7.65 ± 3.55	32,020 ± SEM	11.6 ± 13	92 ± 285	3.34 ± 14.5	59.9 ± 85.4	6.12 ± 4.42	7.6 ± 1.83	10.2 ± 14.3	19.2 ± 7.54
BA.2.86	4,940 ± 1,470	573 ± 302	2,750 ± 3,630	514 ± 227	492 ± 244	285 ± 143	125 ± 66.3	23.9 ± 12.2	72.8 ± 40	16.4 ± 5.26
BA.4/BA.5	5.09 ± 2.12	4,180 ± 24,200	21.3 ± 1,360	105 ± 86.5	0.93 ± 0.39	97.7 ± 18.5	15.6 ± 46.7	10 ± 7.32	12.8 ± 9.99	6.1 ± 3.8
XBB.1.5	>20,000	>20,000	>20,000	16,300 ± 7,310	6,400 ± 1,730	>20,000	2,160 ± 802	77.2 ± 33.9	1,310 ± 777	42.8 ± 15.3
XBB.1.16	>20,000	>20,000	13,000 ± 23,200	>20,000	10,500 ± 32,100	>20,000	2,460 ± 919	24.9 ± 7.95	7,020 ± 32,700	42.9 ± 11.2
EG.5.1	>20,000	>20,000	18,800 ± 9,370	19,400 ± 30,400	10,800 ± 31,300	5,150 ± 268	1,080 ± 1,860	41 ± 32.2	679 ± 263	28.7 ± 8.97
HV.1	>20,000	>20,000	>20,000	>20,000	2,220 ± 3,170	11,900 ± 4,400	3,740 ± 9,220	288 ± 2,230	770 ± 526	31 ± 12.2

Supplemental Table 4. *In vitro* pseudovirus neutralization assay IC_{50} and IC_{90} values for monoclonal antibodies and CoV2-biRNs. Related to Figures 1 and 4.
